# A Case of MDMA-Associated Cerebral and Pulmonary Edema Requiring ECMO

**DOI:** 10.1155/2017/6417012

**Published:** 2017-11-15

**Authors:** A. Thakkar, K. Parekh, K. El Hachem, E. M. Mohanraj

**Affiliations:** ^1^Department of Internal Medicine, Mount Sinai St. Luke's-West Hospital, New York, NY, USA; ^2^Division of Pulmonary, Critical Care & Sleep Medicine, Mount Sinai St. Luke's-West Hospital, New York, NY, USA; ^3^Division of Nephrology, Mount Sinai St. Luke's-West Hospital, New York, NY, USA

## Abstract

A 20-year-old female presented with confusion, generalized tonic-clonic seizures, and severe hyponatremia after ingesting 3,4-methylenedioxymethamphetamine (MDMA). Brain computed tomography (CT) demonstrated cerebral edema. Her hospital course was rapidly complicated by respiratory failure and shock requiring intubation and vasopressors. Refractory acute respiratory distress syndrome (ARDS) was diagnosed which was unresponsive to conventional and salvage therapies, requiring initiation of extracorporeal membrane oxygenation (ECMO), leading to normalization of oxygenation parameters. Hyponatremia was corrected and the encephalopathy resolved. The patient was decannulated and extubated after three days. MDMA-induced hyponatremia is hypothesized to result from enhanced serotonergic activity and arginine vasopressin (AVP) release in the brain leading to hyperthermia-induced polydipsia and syndrome of inappropriate antidiuretic hormone (SIADH) secretion. A common but often unrecognized complication of severe hyponatremia is the Ayus-Arieff syndrome where cerebral edema causes neurogenic pulmonary edema via centrally mediated increases in catecholamine release and capillary injury. For our patient, ECMO was required for three days while the hyponatremia was corrected which led to rapid clearing of the cerebral edema and neurogenic pulmonary edema. This case illustrates that, in selecting patients with refractory ARDS from MDMA-associated cerebral and pulmonary edema, ECMO may be a temporizing and life-saving modality of treatment.

## 1. Introduction

MDMA (3,4-methylenedioxymethamphetamine), a synthetic substance initially patented as an appetite inhibitor or tranquilizer, is now a frequently used recreational drug. Common street names for the drug are* ecstasy* or* molly*. The drug has become popular amongst teenagers and young adults as it is marketed as a “safe” drug without long-term side effects and lack of addiction tendencies. What is not widely advertised is that MDMA has potentially life-threatening acute and chronic effects on several organ systems. There are reports of fatalities associated with MDMA related to hyperpyrexia, rhabdomyolysis, cardiac arrhythmias, hepatic necrosis, cerebrovascular accidents, and drug-related accidents or suicide [1]. We present the case of a young female who ingested MDMA and presented with confusion, severe hyponatremia, seizures, and rapid progression to acute respiratory distress syndrome (ARDS) requiring mechanical ventilation and extracorporeal membrane oxygenation (ECMO).

## 2. Case

A 20-year-old healthy female presented to the emergency room with confusion, vomiting, and generalized tonic-clonic seizure. She was last seen in her usual state of health 12 hours prior to arrival. Friends reported that she consumed an unknown amount of alcohol and ingested a quarter tablet of MDMA. The patient subsequently became paranoid, attempted to climb up walls, and drank ten bottles of water. She had a witnessed generalized tonic-clonic seizure with frothing at the mouth and recurrent seizure en route the Emergency Department. Both seizures broke spontaneously. Her initial vital signs were notable for a temperature of 38.0 degrees Celsius, heart rate of 88 beats/minute, blood pressure of 140/70 mm Hg, respiratory rate of 14 per minute, and oxygen saturation of 97% on room air. The patient was obtunded, and pupils were dilated, equal, and reactive to light bilaterally. Mucus membranes were moist. Neck was supple. Lung auscultation demonstrated good air entry with bilateral rhonchi. Cardiovascular examination was normal without any murmurs, rubs, or gallops. Abdominal exam was normal. Neurologic examination was limited but the patient had normal extremity tone, hyporeflexia was noted in biceps, triceps, knees, and ankles bilaterally, clonus was absent, and normal bilateral Babinski reflexes were noted. She received 10 mg of IV lorazepam for additional seizures and was intubated for airway protection. A summary of relevant laboratory findings is mentioned in Table 1. Her initial complete blood count had a white blood cell count of 20,600/*μ*liter, hemoglobin of 12.8 g/dl, and platelet count of 233,000/*μ*liter. Her chemistry panel was as follows: sodium 112 mmol/L, potassium 3.5 mmol/L, chloride 84 mmol/L, bicarbonate 16 mmol/L, blood urea nitrogen of 7 mg/dl, serum creatinine of 0.5 mg/dl, and serum glucose of 117 mg/dl. A liver function panel was normal.

Serum lactic acid was 2.8 and osmolality was 239 mmol/L. Urine chemistries were significant for a urine sodium 112 mmol/L and urine osmolality of 439 mmol/L. The urine electrolytes were checked prior to administration of any hypertonic saline. These electrolyte derangements suggested a state of syndrome of inappropriate antidiuretic hormone (SIADH). She received three doses of 3% hypertonic normal saline without a significant change in her serum sodium. A brain computed tomography (CT) scan showed cerebral edema.

Over the next couple of hours, she had increasing oxygen requirements on the ventilator of up to 100% FiO2 and a positive end-expiratory pressure of 20 mm Hg. The initial chest-radiograph was concerning for multifocal pneumonia; however a repeat chest-radiograph, twelve hours later, revealed diffuse bilateral hazy opacities concerning for acute respiratory distress syndrome (ARDS). Her blood gas analysis showed a pH of 7.28 with PaO2 of 53 mm Hg and an alveolar-arterial oxygen gradient of 492 mm Hg. Her PaO2 : FiO2 ratio was 53 suggesting severe ARDS. At that time, she was paralyzed with cis-atracurium and started on inhaled nitric oxide as salvage therapy. She subsequently developed hemodynamic compromise and was started on norepinephrine, ultimately requiring addition of vasopressin and dopamine to maintain stable hemodynamics. Finally, venovenous extracorporeal membrane oxygenation (ECMO) was started with immediate resolution of hypoxemia. Hyponatremia was corrected gradually with 3% hypertonic saline that led to resolution of altered mental status and improvement in hypoxemia. She was gradually weaned off of ECMO and extubated within three days.

## 3. Discussion

MDMA is a synthetic compound, which is usually marketed in the form of pills and is taken orally. The drug is readily bound to tissues and metabolized by CYP2D6 and a few other enzymes. These enzymes have a very high affinity and get saturated at low concentrations of the drug, thereby greatly increasing the concentration of MDMA in brain with small increase in drug dosage [1]. The drug exerts effects on all systems of the body, most pronounced in the central nervous system. There is evidence that MDMA leads to local increase in the concentration of monoamine neurotransmitters (norepinephrine, serotonin, and, to an extent, dopamine) at the axon terminals. MDMA blocks the reuptake of serotonin by blocking the serotonin receptor, thereby increasing the local concentration of serotonin. Serotonin affects the thermostat and leads to increased body temperature coupled with increased activity leading to profuse sweating. This triggers intense thirst and the individual tends to drink lots of water. However, studies have demonstrated that ecstasy also independently increases the amount of arginine vasopressin (AVP) in the brain, leading to a state of SIADH, leading to increased water retention by the kidneys. Combined, these two pathological mechanisms lead to hyponatremia which can lead to cerebral edema and increase the intracranial pressure, which can trigger generalized tonic-clonic seizures and potentially compression of brainstem and cerebellum into the foramen magnum leading to fatal disruption of circulation or respiration. Cardiovascular effects of MDMA are mediated by the norepinephrine surge. Increase in blood pressure is the direct effect, which indirectly leads to major and minor cerebral and retinal hemorrhages. Cardiac arrhythmias have also been reported. Patients who have ecstasy intoxication are often jaundiced. The mechanism of hepatic injury is postulated to be oxidative injury to hepatocytes secondary to excess glutathione consumption by MDMA metabolites. Elevation in liver enzymes is typically seen. Biopsy usually shows nonspecific acute hepatitis. Resolution of hepatic injury occurs within a few days to weeks. The excess activity induced by sympathetic overdrive and hyperpyrexia induced by serotonin leads to rhabdomyolysis which can potentially lead to myoglobinuria and renal failure [1]. Hence, MDMA can lead to multiorgan failure. This has been described in many cases as well [2–4]. Risk of death from MDMA intoxication is very high in these patients given risk of cerebral herniation as well as severe systemic effects as described above. Apart from short-term effects, MDMA also leads to long-term psychiatric and physical effects. Psychiatric disturbances include severe depression, paranoias, memory impairment, and panic attacks. Long-term physical disturbances include bruxism, low sympathetic tone leading to labile blood pressure and heart rates, and neurologic impairment [1].

MDMA-induced hyponatremia has been reported in several cases [3, 5–14]. A complication of severe hyponatremia is Ayus-Arieff syndrome where hyponatremia-induced cerebral edema drives pulmonary edema [14, 15]. The mechanism behind Ayus-Arieff syndrome is postulated as follows: cerebral edema leads to increase in intracranial pressure. This in turn leads to a centrally mediated increase in vascular permeability and catecholamine release causing capillary injury. This effect seems to be most pronounced in the pulmonary vasculature. Pulmonary artery hypertension and plasma leakage through injured capillaries result in pulmonary edema and subsequent acute respiratory distress syndrome (ARDS) [15]. This phenomenon has also been described in marathon runners who develop hyponatremia secondary to dehydration and develop pulmonary edema, frequently requiring mechanical ventilation [14]. Our patient presented with symptomatic hyponatremia from MDMA-induced SIADH and primary polydipsia leading to Ayus-Arieff syndrome and resultant severe ARDS with PaO2 : FiO2 ratio of 53 refractory to conventional management and salvage therapies. She responded instantaneously to venovenous ECMO, a modality associated with medical and mechanical complications and increased mortality ratios in some patient groups (elderly individuals and patients with underlying hematologic malignancies) [16]. In this patient, it proved instrumental as a bridge therapy [17]. ECMO gave us the opportunity to correct the underlying hyponatremia which helped clear the pulmonary edema and resolution of ARDS and saved our patient's life.

## Figures and Tables

**Table 1:** Laboratory values.

Relevant labs	Arterial blood gas		Others
	Pre-ECMO	Post-ECMO	
Serum sodium 112 mmol/L	pH 7.28	pH 7.42	Urine toxicology-positive for amphetamines
Urine sodium 112 mmol/L	pO2 53 mm Hg	pO2 99 mm Hg	Serum hCG < 2.39 mIU/ml
Serum osmolality 239 mmol/L	pCO2 41.6 mm Hg	pCO2 23.0 mm Hg	Serum creatinine kinase 1308 IU/mL
Urine osmolality 439 mmol/L	O2 saturation 82.9%	O2 saturation 100%	Troponin (peak) 0.497 mg/mL
Lactic acid 2.8 mmol/L	A-a gradient 492		
TSH 1.8 IU/ml			
